# Supplementary material for: Exploring Community Perspectives on Functional Paediatric Habitual Constipation
Source: Int J Environ Res Public Health. 2024 Aug 2;21(8):1017. doi: 10.3390/ijerph21081017 (PMC11354896; doi:10.3390/ijerph21081017)
Supplement: Supplementary file 1 [file ijerph-21-01017-s001.zip › ijerph-3052485-supplementary.pdf]

International Journal of
Environmental Research and Public Health

MDPI

Supplement Documents:

1. Questionnaire in English:

Questionnaire: Paediatric Functional Constipation

Demographic				
**Gender:**	Male	Female		
**Age:**	20-29 years old	30-39 years old	Over 40 years old	
**Educational Level:**	Uneducated	Less than a bachelor's degree	Bachelor's degree	Postgraduate degree
**Number of Children:**	None	1	2	3 or more

Constipation in General							
**Constipation is more common in:**	Male	Female	Both equally	I don't know			
**Delayed passage of stool during the first 24 hours after birth is a sign of an organic problem in the intestines:**	Yes	No					
**Which of the following statements are correct regarding constipation in children?**							
**Infrequent bowel movements (once every 3 days or more)**	Correct	Incorrect					
**Increased stool hardness and change in shape**	Correct	Incorrect					
**Reduced stool quantity**	Correct	Incorrect					
**Severe pain and difficulty in defecation**	Correct	Incorrect					
**Frequent bloating and increased gas output**	Correct	Incorrect					
**Do delivery methods affect constipation in children:**	Constipation increases with vaginal delivery	Constipation increases with cesarean delivery	There is no difference				
**Does constipation in children relate to health problems:**	Yes	No					
**If Yes, please specify:**	Formula milk	Hereditary causes	Maldigestion / Malabsorption	Neurological disorders	Malnutrition	Anorectal disorders	Respiratory diseases

Constipation and Feeding					
**Does breastfeeding reduce the chances of your child experiencing constipation:**	Yes	No			
**What is the optimal age to wean your child to prevent constipation:**	6-9 months	9-12 months	12-15 months	15-24 months	More than 24 months
**What is the best food for weaning your child?**					
**Natural vegetables and fruits**	Yes	No			
**Yogurt**	Yes	No			
**Ground grains (e.g., cereals)**	Yes	No			
**Boiled meat**	Yes	No			

1

**Commercial baby food**	Yes	No			
**Your child needs to drink at least 5 cups of water daily (one cup equals 200 ml):**	Strongly agree	Agree	Neutral	Disagree	Strongly disagree
**What kind of food does your child over two years old eat?**					
**Food containing fats and carbohydrates such as rice and pasta**	Yes	No			
**A balanced diet containing fiber in vegetables and fruits**	Yes	No			
**Eating yogurt and milk**	Yes	No			
**Sweets and children's desserts**	Yes	No			
**How often do you allow your child to eat sweets and desserts:**	More than once a day	Once a day	3-6 times a week	Less than twice a week	

**Constipation and Toilet**					
**I will start by stopping the use of diapers for my children at the age of:**	12 months or less	12-15 months	15-24 months	24-36 months	More than 36 months
**How often does your child have a bowel movement in the toilet:**	Every day	3 to 4 times a week	Once or twice a week	Less than once a week	
**Does your child use any aids to help with bowel movements:**	Yes, medication/suppositories	Yes, natural remedies/products	No		
**Does your child suffer from or feel pain during bowel movements:**	Always	Often	Sometimes	Never	
**Does the child refuse to use the toilet outside the home (public places and school) for bowel movements:**	Always	Often	Sometimes	Never	
**Do the child's clothes get soiled with stool when they suffer from constipation:**	Always	Often	Sometimes	Never	

**Constipation and Behavior**					
**The child may hide their soiled clothes due to chronic constipation:**	Strongly agree	Agree	Neutral	Disagree	Strongly disagree
**Constipation can cause a decrease in the child's appetite or weight loss:**	Strongly agree	Agree	Neutral	Disagree	Strongly disagree
**Constipation can affect your child's academic performance:**	Strongly agree	Agree	Neutral	Disagree	Strongly disagree
**Constipation can affect your child's behavior:**	Strongly agree	Agree	Neutral	Disagree	Strongly disagree
**Constipation can negatively affect your child's overall health:**	Strongly agree	Agree	Neutral	Disagree	Strongly disagree

2

2. Questionnaire in Arabic:

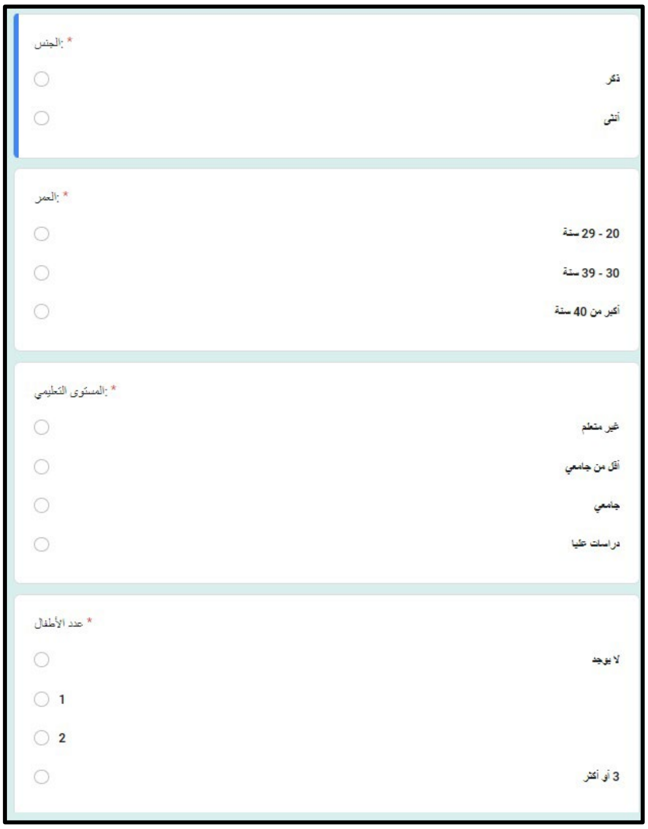
الجنس: *
ذكر
أنثى
العمر: *
20 - 29 سنة
30 - 39 سنة
أكبر من 40 سنة
المستوى التعليمي: *
غير متعلم
أقل من جامعي
جامعي
دراسات عليا
عدد الأطفال *
لا يوجد
1
2
3 أو أكثر

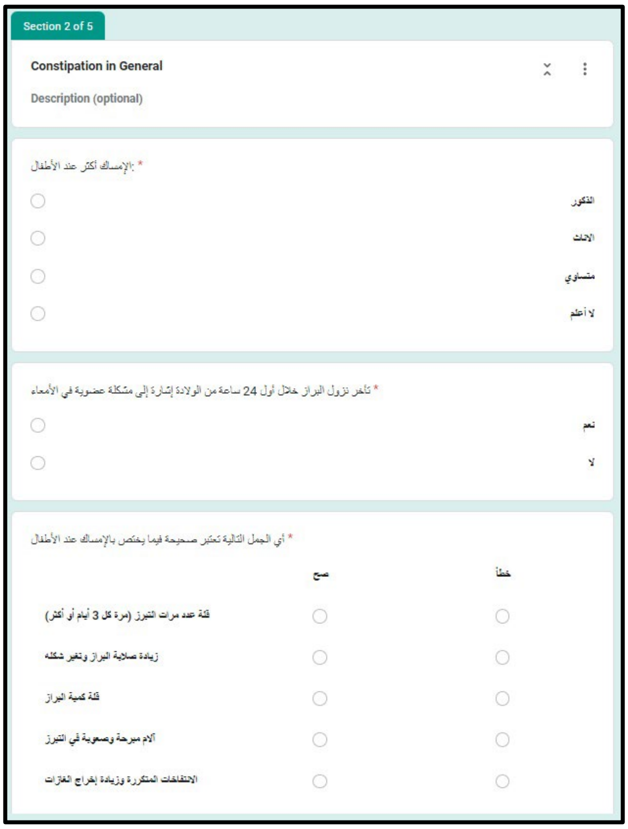
Section 2 of 5
Constipation in General
Description (optional)
الإمساك أكثر عند الأطفال: *
الذكور
الاناث
متساوي
لا أعلم
تأخر نزول البراز خلال أول 24 ساعة من الولادة إشارة إلى مشكلة عضوية في الأمعاء *
نعم
لا
أي الجمل التالية تعتبر صحيحة فيما يختص بالإمساك عند الأطفال *

	صح	خطأ
قلة عدد مرات التبرز (مرة كل 3 أيام أو أكثر)		
زيادة صلابة البراز وتغير شكله		
قلة كمية البراز		
آلام مبرحة وصعوبة في التبرز		
الانتفاخات المتكررة وزيادة إخراج الغازات		

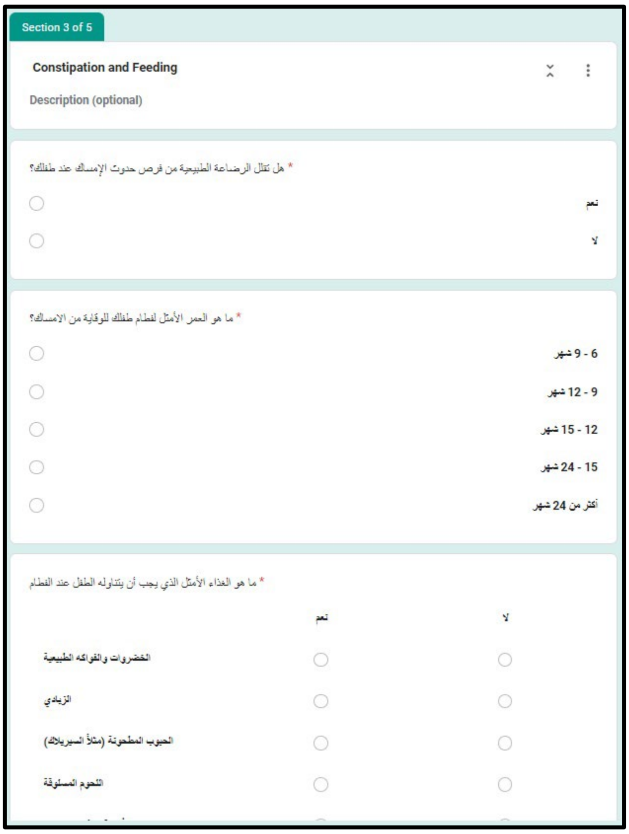
Section 3 of 5

Constipation and Feeding

Description (optional)

هل تقلل الرضاعة الطبيعية من فرص حدوث الإمساك عند طفلك؟ *

- نعم
- لا

ما هو العمر الأمثل لفطام طفلك للوقاية من الامساك؟ *

- 6 - 9 شهر
- 9 - 12 شهر
- 12 - 15 شهر
- 15 - 24 شهر
- أكثر من 24 شهر

ما هو الغذاء الأمثل الذي يجب أن يتناوله الطفل عند الفطام *

	نعم	لا
الخضروات والفواكه الطبيعية	○	○
الزبادي	○	○
الحبوب المطحونة (مثلاً السيريلاك)	○	○
اللحوم المسلوقة	○	○

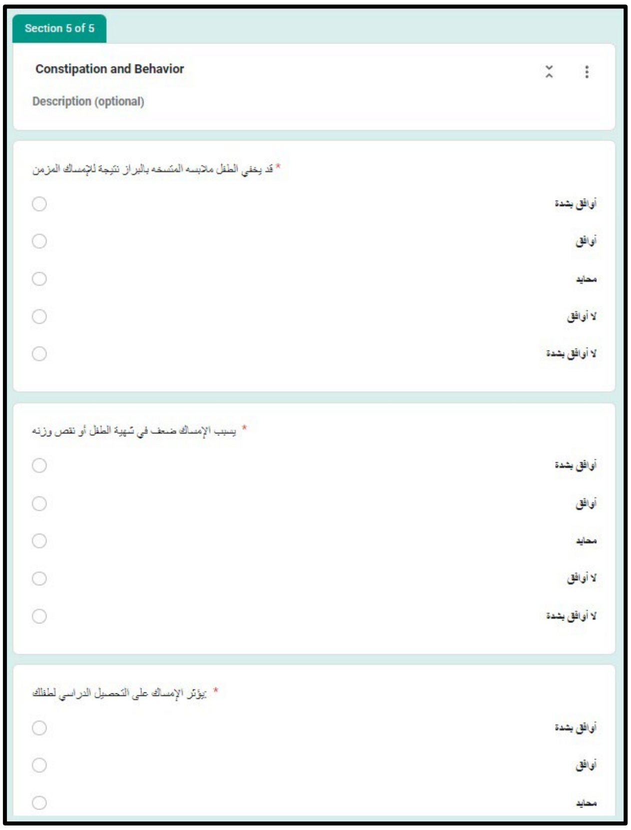
Section 5 of 5

Constipation and Behavior

Description (optional)

قد يخفي الطفل ملابسه المتسخه بالبراز نتيجة للإمساك المزمن *

- أوافق بشدة
- أوافق
- محايد
- لا أوافق
- لا أوافق بشدة

يسبب الإمساك ضعف في شهية الطفل أو نقص وزنه *

- أوافق بشدة
- أوافق
- محايد
- لا أوافق
- لا أوافق بشدة

يؤثر الإمساك على التحصيل الدراسي لطفلك *

- أوافق بشدة
- أوافق
- محايد
